# Cell-cycle arrest and senescence in TP53-wild type renal carcinoma by enhancer RNA-P53-bound enhancer regions 2 (p53BER2) in a p53-dependent pathway

**DOI:** 10.1038/s41419-020-03229-8

**Published:** 2021-01-05

**Authors:** Haibiao Xie, Kaifang Ma, Kenan Zhang, Jingcheng Zhou, Lei Li, Wuping Yang, Yanqing Gong, Lin Cai, Kan Gong

**Affiliations:** 1 Department of Urology, Peking University First Hospital, Beijing, People’s Republic of China; 2 Institute of Urology, Peking University, Beijing, People’s Republic of China; 3 National Urological Cancer Center, Beijing, People’s Republic of China; 4 Hereditary Kidney Cancer Research Center, Peking University First Hospital, Beijing, People’s Republic of China

**Keywords:** Tumour biomarkers, Renal cell carcinoma

## Abstract

TP53 is a classic tumor suppressor, but its role in kidney cancer remains unclear. In our study, we tried to explain the role of p53 in kidney cancer through the p53-related enhancer RNA pathway. Functional experiments were used to explore whether P53-bound enhancer regions 2 (p53BER2) has a role in the cell cycle and senescence response of TP53-wild type (WT) renal cancer cells in vitro or vivo. RNA-sequencing was used to identify the potential target of p53BER2. The results showed that the expression level of P53BER2 was downregulated in renal cancer tissues and cell lines, further dual-luciferase experiments and APR-256-reactivated experiments showed p53BER2 expresses in a p53-dependent way. Moreover, knockdown p53BER2 could reverse nutlin-3-induced cytotoxic effect in TP53-WT cell lines. Further exploration showed the downregulation of p53BER2 could reverse nutlin-3-induced G1-arrest and senescence in TP53-WT cell lines. What is more, the knockdown of p53BER2 showed resistance to nutlin-3 treatment in vivo. Additionally, we found BRCA2 could be regulated by p53BER2 in vitro and vivo; further experiment showed p53BER2 could induce cell-cycle arrest and DNA repair by mediating BRCA2. In summary, the p53-associated enhancer RNA-p53BER2 mediates the cell cycle and senescence of p53 in TP53-WT renal cancer cells.

## Introduction

Kidney cancer is one of the most common tumors of the urinary system^1^. According to GLOBOCAN guideline 2018, there were 403,262 new renal cancer cases and 175,098 death cases for renal cancer worldwide in 2018^1^. In the U.S., the corresponding number of the above cases in 2014 was 63,290 and 13,860^2^. In the past decade, the incidence of kidney cancer has been steadily rising. Except for improvements in cancer screening and diagnostic technologies, one important reason is the lack of an effective and sustainable treatment strategy. Traditional chemotherapy and radiotherapy usually function well in epithelial cancers, but it seems relatively resistant in renal cancer^3^. To address this problem, many scientists focus on signaling pathway about chemotherapy resistance. And one of the most famous pathways that causing therapy resistance to epithelial cancers is the inactivation of the p53 signaling pathway^4^.

The tumor protein p53 (TP53) gene is the most common mutant gene in human cancers, in which mutations occur in more than 50% of tumors. In the case of tumors carrying the wild-type TP53 gene, changes in other p53-pathway components are believed to be responsible for its inactivation^5^. The p53 protein, encoded by the TP53 gene, is a transcription factor that can regulate the expression of multiple target genes to mediate the diverse cellular responses, such as apoptosis, cell-cycle arrest, and DNA repair^6^. Although p53 has a very important role in the tumor, its’ function in renal cancer remains unclear. In many cancers, high-level expression of p53 is used as a surrogate indicator of TP53 mutation due to the instability of MDM2/p53 homeostasis^7,8^. But this is not the case in kidney cancer. Firstly, the interpretation is not fit in the renal cancer line, the TP53-WT renal cancer line could express p53 at a relatively high level^9^. Additionally, one interesting study from Chemeris et al showed that none of the 29 RCC samples with positive staining of p53 had a TP53 point mutation^10^. Actually, even if p53 could be stained positively in 24.5% of RCC samples^11^, the overall TP53 mutation ratio in RCC samples was lower than 10%^12,13^. It appears likely that p53 upregulation is not correlated with TP53 mutation in most RCC samples, which means another factor might block the p53 pathway in kidney cancer. And this tissue-specific genotype and phenotype correlation might provide a chance for reactivating p53 function to kill cancer cells. Some groups have found that reactivating p53 by inhibiting MDM2, which is an E3 ligase for p53, could repress the proliferation of renal cancer cells^14,15^. But the specific mechanism of how to reactivate p53 function in kidney cancer remains unclear.

Enhancer RNAs (eRNAs) is a kind of novel long non-coding RNA, which is encoded by some enhancer sites^16^. Under some special situation, RNA polymerase II (RNAPII) will bind to specific enhancer sites to produce eRNAs^17^. It was believed that the expression of eRNAs related to the mRNA level of surrounding protein-coding genes^18^. What is more, recent studies have revealed that eRNAs have an important role in cancers^19,20^. For instance, 17 β-oestradiol (E2)-bound estrogen receptor α (ER-α)-related eRNAs could induce the expression of target coding genes by increasing the binding of specific enhancer-promoter looping with ER-α^19^. Recently, it was found that p53 could regulate the non-coding genomic regions to produce eRNAs as well^21^. P53-bound enhancer regions (p53BERs) produce eRNAs, which are required for efficient transcriptional of target genes to exert p53’s function^22^. Since the function of p53 in renal cancer cannot be fully explained from the protein’s perspective^23^, we aimed to figure out whether eRNAs have an important role in p53 pathway in renal cancer.

In this study, we included some p53-related eRNAs and tested them in our clinical renal cancer sample. We demonstrated that p53BER2 is extremely low in renal cancer compared with normal kidney tissue. Further, we show that p53BER2 is a functional eRNA involved in the regulation of the cell cycle and senescence in renal cancer cells. Therefore, our results suggested that p53BER2 mediates the cell cycle and senescence of p53 in TP53-WT renal cancer cells. This further provides a new research approach for the molecular mechanism of renal cancer.

## Materials and methods

### Patients and clinical renal carcinoma samples collections

A total of 84 fresh kidney cancer samples and pair-matched normal adjacent renal tissue were collected in Peking University first hospital from 2015 to 2019. All samples were stored in RNA-later Stabilization Solution (Invitrogen, US) before extraction and were transferred to liquid nitrogen immediately. The main feature of the included patients was shown in Supplementary Table 1. All the patients have signed informed consent included a statement confirming that consent to publish after understanding the process consequences of the study and the project was approved by the Medical Ethics Committee of Peking University First Hospital (Beijing, China).

### Cell lines and transfection and infection

RCC cell lines Caki-1, 786-O, ACHN, OSRC-2, H1299, and the normal tubular epithelial cell line HK-2 was purchased from the American Type Culture Collection (ATCC, Manassas, VA). HK-2 cells were cultured in DMEM/F12 medium containing 10% fetal bovine serum (HyClone Laboratories Inc., Logan, UT), 786-O, H1299, and OSRC-2 were cultured in RPMI-1640 (HyClone, Logan, UT) medium. Plus add 10% GibcoTM FBS (Life Technologies, Grand Island, NY). caki-1 and ACHN were cultured in DMEM (HyClone, Logan, UT) medium plus 10% GibcoTM FBS (Life Technologies, Grand Island, NY). All cells were incubated at 37 °C in a standard humidified incubator containing 5% CO_2_ and 95% O_2_. The MDM2 inhibitor nutlin-3 (Selleck S1061, US) and PRIMA-1-met (Sigma-Aldrich, SML1789) were added according to a different cell and experimental conditions. Both of these two drugs treated the cell for 24 h before collecting the cell and conducting the experiments.

AmicroRNA vectors and “p53BER2 reporters” were bought from Beijing Syngentech Co, Ltd, pLX313-TP53-WT and pLX313-TP53-P278A were bought from Addgene. The CDS sequence of BRCA2 was cloning in a pcDNA 3.1 backbone. For transfection, RCC cell lines were seeded on 6-well plates at 3 × 10^5^ cells/well (~90–95% confluency). Cells were transfected the following day with Lipofectamine 3000 transfection reagent (L3000008, ThermoFisher Scientific). Cells were harvested 48 h after transfection. For the construction of stable cell lines, 293T packaging cell lines were used for lentiviral amplification. Lentiviral infection was carried out as previously described^24^. Briefly, viruses were collected at 48 and 72 h post-transfection. After passing through 0.45-μm filters, viruses were used to infect target cells in the presence of 8 μg/mL polybrene. Subsequently, target cell lines underwent appropriate antibiotic selection.

### RNA isolation and quantitative real-time polymerase chain reaction (qRT-PCR)

Extraction of total RNA from cells were conducted by TRIzol reagent (Invitrogen, USA) according to manufacturer’s instruction. Reverse transcription total RNA was performed using RevertAid First Strand cDNA Synthesis Kit (ThermoFisher Scientific, USA). RT-qPCR was performed using a standard SYBR Green PCR Kit (ThermoFisher Scientific, USA) on an ABI 7500 PCR System (Applied Biosystems, Foster City, CA, USA) according to the manufacturer’s instructions. Relative expression levels were calculated using the 2^−ΔΔCt^ method. The transcription level of GAPDH serves as an internal reference. Supplementary Table 4 lists artificial microRNA vector and specific primer sequences.

### Western blotting and immunofluorescence

The method of extracting total protein from cultured cells and specimens was to use RIPA buffer (Applygen, China) according to the manufacturer’s instructions. SDS-PAGE and Western blot were performed according to standard protocols. Immunoreactive bands were visualized by Pierce Fast Western Blot Kit (ThermoFisher, USA) using an SYNGENE G: BOX imaging system (Frederick).

For immunofluorescence, we seeded stable RCC cells in 24-well plates. 4% formaldehyde was used to fix the cells (~70–90% confluency) for 15 min at room temperature. PBS wash three times for 5 min each. Block specimen in Blocking Buffer for 60 min. Aspirate blocking solution, apply diluted primary antibody. Incubate overnight at 4 °C. Rinse three times in PBS for 5 min each. Incubate specimen in fluorochrome-conjugated secondary antibody diluted in Antibody Dilution Buffer for 1–2 h at room temperature in the dark. Rinse three times in PBS for 5 min each. Coverslip slides with DAPI for 5 min. The images were captured by a Confocal microscope (Leica, Heidelberg, Germany). All of the above solutions were included in Immunofluorescence Application Solutions Kit (Cell signaling technology, #12727, USA). The antibody conjugated to the primary antibody, and the secondary HRP is described in Supplementary Table 4.

### Dual-luciferase reporter assay

To directly figure out the interaction between p53 and p53BER2, a dual-luciferase reporter assay was performed using p53BER2 constructs (Fig. 2C). The p53BER2 reporter contained copies of p53BER2-binding sites, a minimal promoter, and dual-reporter vector (Beijing Syngenetech CO., Ltd, China). cDNAs of the binding sites of p53BER2 are shown in Supplementary Table 4. Cells were seeded into six-well plates (5 × 10^5^ per well) and transfected with p53BER2 reporter vectors. Luciferase activity was measured using the dual-luciferase assay system (Promega, Madison, WI, USA) as per the manufacturer’s instructions at 48 h after transfection. Firefly luciferase activities were normalized to Renilla luciferase activities. All assays were performed in duplicate, and all data shown are representative of at least two independent experiments.

### RNA fluorescent in situ hybridization (RNA-FISH)

The FISH assay was performed using Ribo™ Fluorescent in Situ Hybridization Kit (Ribobio Company, China). P53BER2, 18S, and U6 probes were designed and synthesized by Ribobio Company and labeled with Cy3 fluorescent dye. RNA-FISH was performed using fluorescent in situ hybridization kit (RiboBio) following the manufacturer’s instructions. Fluorescence detection was performed with a confocal laser-scanning microscope (Leica, Heidelberg, Germany). 18S and U6 were used as cytoplasm and nuclear reference.

### In vitro cell proliferation assay

Cell proliferation was determined using Cell Counting Kit-8 (TransGen Biotech, China) according to the manufacturer’s instructions. Briefly, 5 × 10^3^ cells/well were seeded in a 96-well flat-bottomed plate, and grown at 37 °C for 24 h, then transfected with the corresponding vector, or incubated with nutlin-3. Finally, the absorbance was finally determined at a wavelength of 450 nm using a microplate reader (Bio-Rad, Hercules, CA, USA). Experiments were repeated at least three times.

Cell proliferation was also determined by Ethynyl-2- deoxyuridine incorporation assay using an EdU Apollo DNA in vitro kit (RIBOBIO, Guangzhou, China) following the manufacturer’s instructions. Briefly, after transfected with corresponding vector cells were incubated with 100 μl of 50 μM EdU per well for 2 h at 37 °C, respectively. Finally, the cells were visualized under fluorescence microscopy. Experiments were repeated at least three times.

### In vivo tumor growth

RCC cell-derived xenograft (CDX) models were established as previously described^25^. Briefly,5*106 stable RCC cells were injected into the axilla of 5-weeks BALB/c male nude mice randomly (Vital River Laboratory Animal Technology Co., Ltd.). When the length of the tumor size reached over 2 mm, the volume size would be collected twice per week. When tumors reached a mass of ~50 mm^3^, mice were treated with Nutlin-3 (20 mg/kg) every 2 days. Tumor samples were resected when the length of tumor size reaches 2 cm. Technician conducted the tumor cell and drug injection without telling the groups of the tumor cell. Animal experiments were conducted according to the Guideline of the Care and Use of Laboratory Animals in Peking University First Hospital and were approved by the Medical Ethics Committee of Peking University First Hospital (Beijing, China).

### Flow cytometry

Cell apoptosis was assayed by staining with Annexin V-APC and PI (KeyGEN BioTECH) following the manufacturer’s instructions and detected by a flow cytometer (FACSCalibur, Becton Dickinson, New Jersey, USA).

### Cell-cycle assay

For the cell-cycle assay, DATS (Diallyl trisulfide)-treated cells were harvested, washed twice in phosphate-buffered saline (PBS), and fixed in 75% cold alcohol overnight at 4 °C. After washing in cold PBS three times, cells were incubated with 1 × PI/RNase staining buffer for 15 min in the dark at room temperature. Samples were then analyzed for their DNA content using flow cytometry. All assays were performed in duplicate, and all data shown are representative of at least two independent experiments.

### Senescence β-galactosidase staining assay

Cells after 72 h of culture in each group were seeded into a six-well plate, adjusted to a cell density of ∼50% using RPMI-1640 medium. After cell attachment, the culture medium was removed, and the cells were washed once with PBS. In accordance with the instructions of the senescence β-galactosidase Staining kit (Beyotime, China), SA-β-gal (1 ml) was added into cells for incubation overnight at 37 °C. Positive cells presented blue. Five fields were randomly selected and observed under an optical microscope to calculate the number of positive cells.

### High-throughput cDNA sequencing (RNA-seq)

Total RNA was extracted from two RCC cell lines (OSRC-2 and ACHN) treated with recombinant lentivirus overexpressing amicroRNA-p53BER2 or negative control and quantitated using a NanoDrop-1000 spectrophotometer, and the integrity was subsequently assessed with an Agilent 2100Bioanalyzer (Agilent Technologies, Santa Clara, CA, USA). RNA-sequencing was performed on an Illumina HiseqX10. Experiments were repeated three times. For the detection of differentially expressed genes (DEGs), a fold changes ≥ 2 or ≤1/2 and a false discovery rate (FDR) < 0.01 were set as the screening criteria. *P*-values ≤ 0.05 were considered statistically significant. Kyoto Encyclopedia of Genes and Genomes (KEGG) pathway analysis and Gene ontology pathway analysis was used to clarify the physiological functions and signaling pathways related to the DEGs in RCC cell lines.

### Homologous recombination (HR) assay

Host cell reactivation-based HR assay (Norgen Biotek, Thorold, ON, Canada) was performed on RCC cells according to the manufacturer’s instructions. Briefly, on day 2 after nutlin-3 treatment, cells were transfected with a positive control plasmid or two HR dl plasmids (dl-1 and dl-2). After 24 h of transfection, total cellular DNA was isolated. Then, qPCR was performed with the supplied primers using an ABI 7500 PCR System (Applied Biosystems, Foster City, CA, USA).

### Statistical analysis

Each experiment was performed in triplicate. All quantitative data are expressed as mean ± standard deviation (SD). Statistical analyses were performed using SPSS8.0 software (IBM Corp., Armonk, NY, USA). Statistical significance was tested by Student’s *t*-test, chi-square test, or ANOVA. *P* < 0.05 was statistically significant.

## Results

### P53BER2 expression is downregulated in renal cancer tissues and cell lines

**Fig. 1:**
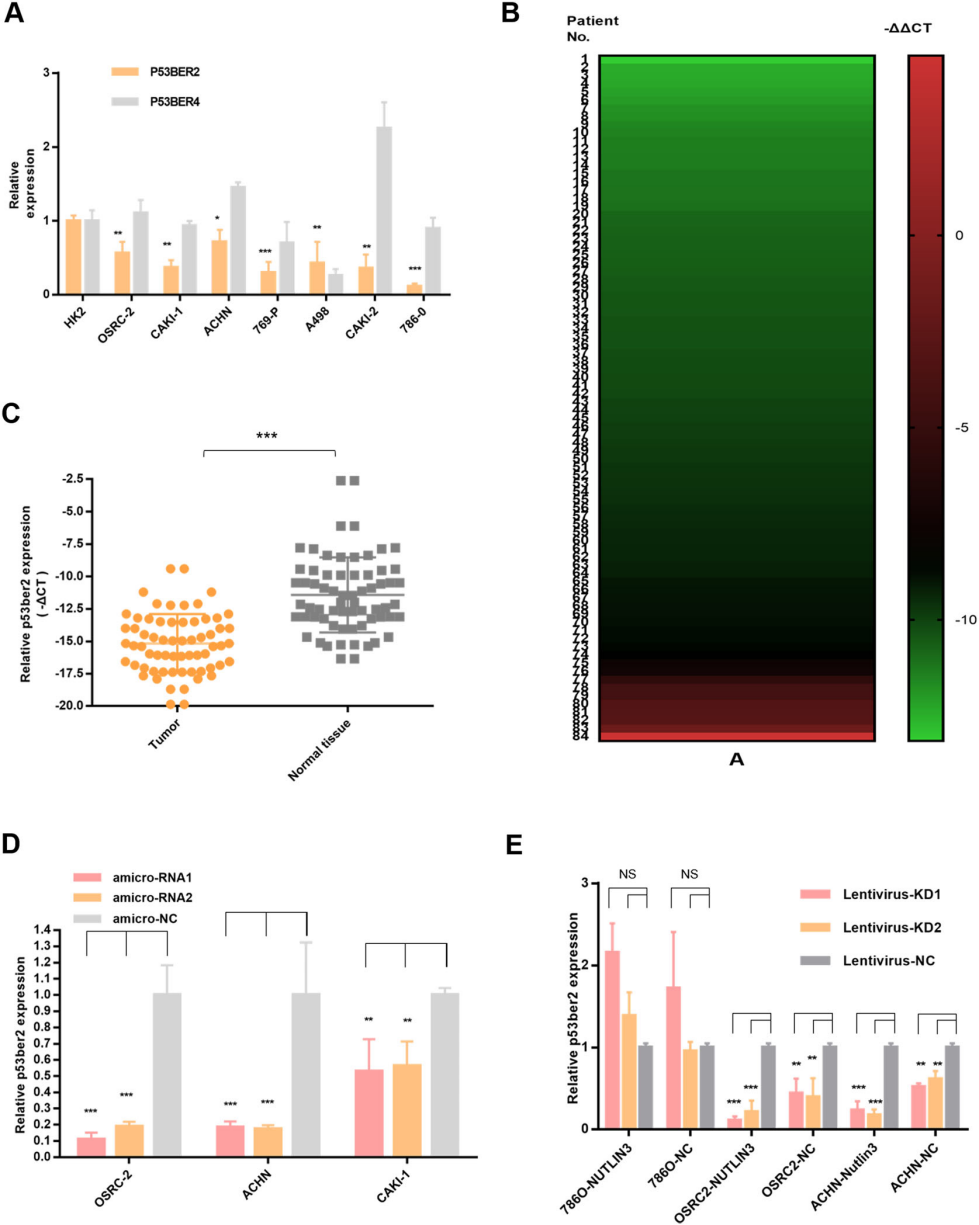
Clinical information and p53-related eRNAs expression level in all the RCC tumor samples and cell lines. **A** P53-related eRNAs expression level in RCC cell lines. **B** The heatmap about the expression level of p53BER2 in 84 RCC tumor samples. **C** The expression of p53BER2 in RCC tumor samples and normal tissues. **D** The relative p53BER2 expression level in different RCC cell lines after transfecting amicroRNA. **E** The relative p53BER2 expression level in different RCC cell lines after infecting lentivirus. Results are shown as mean ± SD. ***P < 0.001 compared with control. **P < 0.01 compared with control. *P < 0.05 compared with control. NS not significantly.

As previously reported, we selected p53-associated enhancer RNA (p53BER2 and p53BER4) for our study. First, we examined the expression of p53BERs in seven renal cell carcinoma cell lines and the renal epithelial cell line (HK2) and found that the expression level of p53BER2 was downregulated in all renal cancer cell lines, relative to the normal renal epithelial cell line (HK2) (Fig. 1A). However, there was no significant difference in p53BER2 levels between renal cell carcinoma and normal renal epithelial cell lines (Fig. 1A). Further, we examined the expression level of p53BER2 in 84 paired kidney cancer tissues and found that the expression level of p53BER2 in kidney cancer was much lower than that in normal kidney tissues (Fig. 1C). Among them, p53BER2 was expressed in 79% (67/84) of renal cancer tissues relative to normal tissues (Fig. 1B). More importantly, we found that p53BER2 predicts a higher tissue grade (*P* = 0.007) in 83 patients (1 with loss of pathology information), accompanied by a trend toward higher-level T staging (Table 1). Detailed patient clinical information and related analysis can be found in Table 1 and Supplementary Table 1.

**Table 1:** Association between p53BER2 expression level and clinical features of RCC patients.

Variable	Patients	P53BER2 (−ΔCT/median)		*χ*^2^	*P*-value
		Low (*n* = 42)	High (*n* = 42)		
Age (years), mean ± SD	54.73 ± 12.24	57.05 ± 12.18	52.48 ± 11.86		
Age (years), *n* (%)				0.764	0.382
≥55	45	20	25		
<55	39	22	17		
Gender, *n* (%)				0.223	0.637
Male	59	29	30		
Female	25	13	12		
T stage, *n* (%)				4.402	0.111
T1a	42	18	24		
T1b + T2	29	14	15		
T3	13	10	3		
Fuhrman grade, *n* (%)				9.973	0.007
G1	30	8	22		
G2	43	26	17		
G3 + G4	11	8	3		

*RCC* renal cell carcinoma, *CT* cycle threshold, Fuhrman nuclear grade.

### P53BER2 could be specifically expressed in TP53-WT renal cancer cell lines

**Fig. 2:**
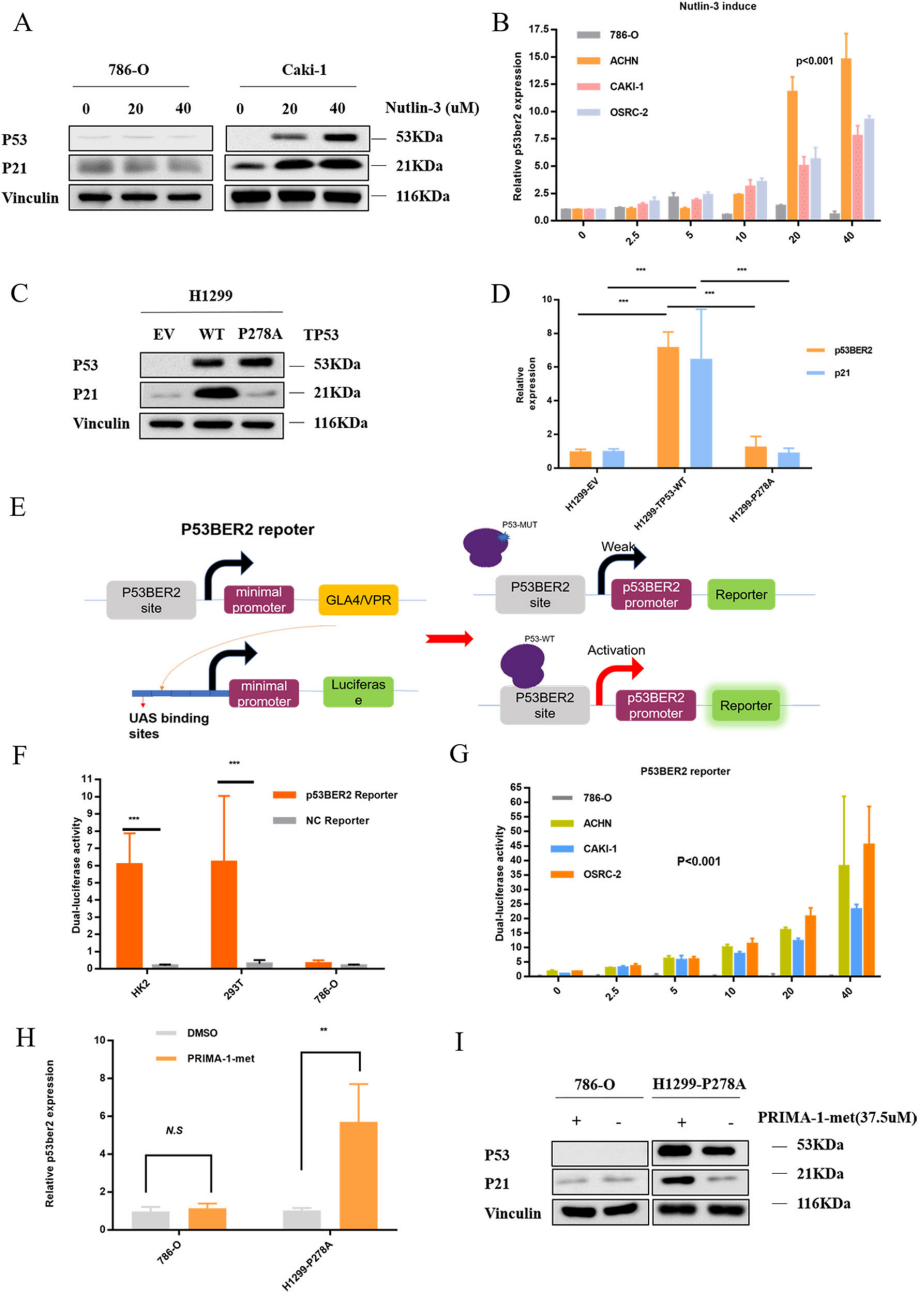
P53BER2 could be induced by MDM2 inhibitor-nutlin3 in TP53-WT RCC cell lines. **A** P53 could be induced by nutlin3 in TP53-WT RCC cell lines. **B** P53BER2 could be induced by nutlin3 in TP53-WT RCC cell lines. **C** Western blot of H1299 after infected by pLX313-TP53-WT and pLX313-TP53-P278A. **D** Exogenous P53 but not P53-278A could induce p53BER2 in H1299. **E** The design and construction of the p53BER2 reporter. **F** The verification of p53BER2 reporter in TP53-WT and p53-MUT cell lines. **G** The dual-luciferase assay showed WT-p53 could induce p53BER2 by binding the p53BER2 promoter. **H** The relative p53BER2 expression level in 786-0 and H1299-p278A after PRIMA-1-met (37.5 μM). **I** Western blot of 786-0 and H1299-p278A after PRIMA-1-met (37.5 uM). EV empty vector. Results are shown as mean ± SD. ***P < 0.001 compared with control. **P < 0.01 compared with control. *P < 0.05 compared with control.

Since p53BER2 is an enhancer RNA mediated by wild-type p53, we wonder whether p53BER2 is mediated in wild-type p53 in renal cancer cells. First, as shown in Fig. 1A, we could find that p53BER2 expression in TP53 mutant cells (786-O) is the lowest, consistent with our conjecture. To further understand the relationship between p53BER2 and wild-type p53 protein, we used the p53 protein activator nutlin3a to treat p53 wild-type and TP53 mutant kidney cell lines. Western Blot showed that nutlin3 was effective in inducing p53 expression and its target- p21 in Caki-1, but not in the 786-O cell line (Fig. 2A). qPCR results indicated that nutlin3a could induce p53BER2 in p53 wild-type cells (OSRC-2, ACHN, CAKI-1), but did not induce TP53 mutant cell expression (Fig. 2B). Also, we included p21 and PUMA as the positive control, and the results showed the expression of p21 and PUMA were upregulated with nutlin-3 treatment (Fig. 2A and Supplementary Fig. 1C, D). But the PAPPA expression, the previous target of p53BER2^22^, did not change with nutlin-3 treatment, which might be due to different biological mechanisms in different tissue and cells (Supplementary Fig. 1E).

To further explore the relationship between p53 and p53BER2, we got the pLX313-TP53-WT and pLX313-TP53-P278A, then we used the corresponding lentivirus and empty vector (EV) lentivirus to infect H1299, which were found as a p53-null cell^26^. Then we used WB and qPCR to test the expression of p53 and p53BER2; the results showed that overexpression of p53 induced an obviously increased expression of p53BER2 in WT-TP53 H1299 cells, but not in the TP53-P278A H1299 (Fig. 2C, D). Then we used si-P53 to transfect H1299-wt-TP53; qPCR results revealed that downregulation of p53 could decrease the expression level of p53BER2 (Supplementary Fig. 2A, B).

### “p53BER2 reporter” could detect the expression of p53BER2 in TP53-wt RCC cell lines

It has been reported in the literature that wild-type p53 can bind to p53BER2 to enhance promoter expression. Using this principle, we designed the p53BER2 reporter to further investigate whether P53 initiates the promoter by direct binding to p53BER2. First, we combined the p53BER2-specific sequence with the minimal promoter to form a “p53 promoter” that specifically recognizes the wild-type p53 protein. Further, we used the GAL4-UAS system to enhance promoter efficiency and use dual to report the gene, and finally form the “p53BER2 reporter” (Fig. 2E). Since 293T and HK2 have a basic expression of wt-p53^27,28^ and p53, 786-O express a relatively low level of mut-p53 protein. Here we transfected the “p53 reporter” and “control” reporters into the HK2, 293T, and 786-O cell lines, respectively, and we found that the p53 reporter can effectively detect wild-type p53 expression (Fig. 2F). Further, we transferred the reporter into p53 wild-type cells treated with nutlin3 and found that p53 wild-type cells can express higher dual-luciferase intensity with increasing nutlin3 concentration (Fig. 2G), suggesting that the “p53BER2 reporter” could be effective. The expression of the p53 wild-type renal cancer cell line p53BER2 was reported, and the results were consistent with the qPCR results. Also, we transfected H1299/WT/P278A with “p53BER2 reporter” and “NC reporter”. As shown in the Supplementary Fig. 2C, H1299-wt-p53 could express a higher dual-luciferase level than the H1299 and H1299-P278A group.

### Reactivation of mut-TP53 could also induce p53BER2

Many studies have shown PRIMA-1-met could reactivate the function of mut-p53^29–31^. So here we treated 786-O (mut-p53-P278A) and H1299-P278A with PRIMA-1-met. The results showed expression of p53BER2 is upregulated after treating with PRIMA-1-met (Fig. 2H) in H1299-P278A, but not in 786-O, which might be due to the relatively low mut-p53 expression level in 786-O (Fig. 2A, I). As a control, P21 is also upregulated by PRIMA-1-met in H1299-P278A, which means the function of mut-p53 is reactivated in H1299-P278A (Fig. 2I). Furthermore, we used PRIMA-1-met to treat H1299-P278A and H1299 cells which have already transfected with “p53BER2 reporter”; the results showed that PRIMA-1-met could induce higher luciferase level in the H1299-P278A group compared with the H1299 group (Supplementary Fig. 2D). These results demonstrate that wild-type P53 can bind p53BER2-specific sequence to promote transcription of p53BER2, thus confirming our conjecture.

### P53BER2 could mediate p53-related function in TP53-WT renal cancer cell lines

#### Knockdown P53BER2 could reverse nutlin-3-induced cytotoxic effect in TP53-WT cell lines

**Fig. 3:**
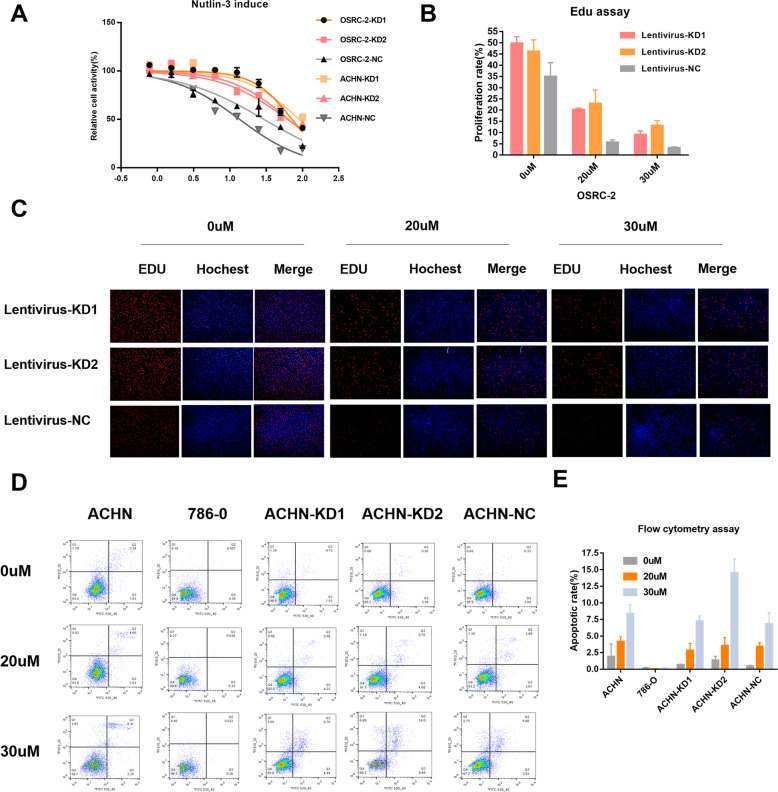
P53BER2 could mediate p53-related function in TP53-WT renal cancer cell lines. **A** The relative cell activity of different stable RCC cell lines after nutlin-3 treatment. **B**, **C** The proliferation rate of OSRC-2 stable cell lines infected amicroRNA or vector after nutlin-3 treatment by EDU assay, scale bar, 100 μm. **D**, **E** The apoptotic rate of different RCC cell lines or stable cell lines after nulin-3 treatment. Results are shown as mean ± SD. ***P < 0.001 compared with control. **P < 0.01 compared with control. *P < 0.05 compared with control.

To explore whether p53BER2 affects p53-related functions. We generated artificial microRNA to knockdown the expression of p53BER2. We found that amicroRNA can effectively knockdown p53BER2 expression (Fig. 1D), so in the subsequent experiments, we constructed the corresponding lentiviral vector using amicroRNA, and further, we constructed the p53BER2 knockdown stable cell lines of OSRC-2 and ACHN-786-O by lentivirus, and The knockdown effect was examined (Fig. 1E) and it was found that amicroRNA can knockdown p53BER2 in the presence of nutlin3 and the absence of nutlin3. To examine the effect of p53BER2 on cell proliferation activity, cck8 assay first showed that nutlin3 could effectively inhibit the proliferation of p53 wild-type renal cell carcinoma cell line (Supplementary Fig. 1A). Interestingly, we found that the knockdown of p53BER2 partially reverses nutlin3-mediated cytotoxic activity (Fig. 3A), accompanied by an elevated IC50 value (Supplementary Fig. 1B). We used the EDU assay to further validates this phenomenon in Fig. 3B, C. To explore why p53BER2 mediates changes in cell proliferation activity, we first hypothesized that p53BER2 affects proliferative activity by mediating cell apoptosis. Using PI/FITC flow cytometry, we found that nutlin3 can induce apoptosis in p53 wild-type renal cell carcinoma cells (Fig. 3D, E), but knocking down p53BER2 does not reverse nutlin3-mediated apoptotic response in ACHN (Fig. 3D) and OSRC-2 (Supplementary Fig. 2E).

#### Knockdown p53BER2 could reverse nutlin-3-induced G1-arrest and senescence in TP53-WT cell lines

**Fig. 4:**
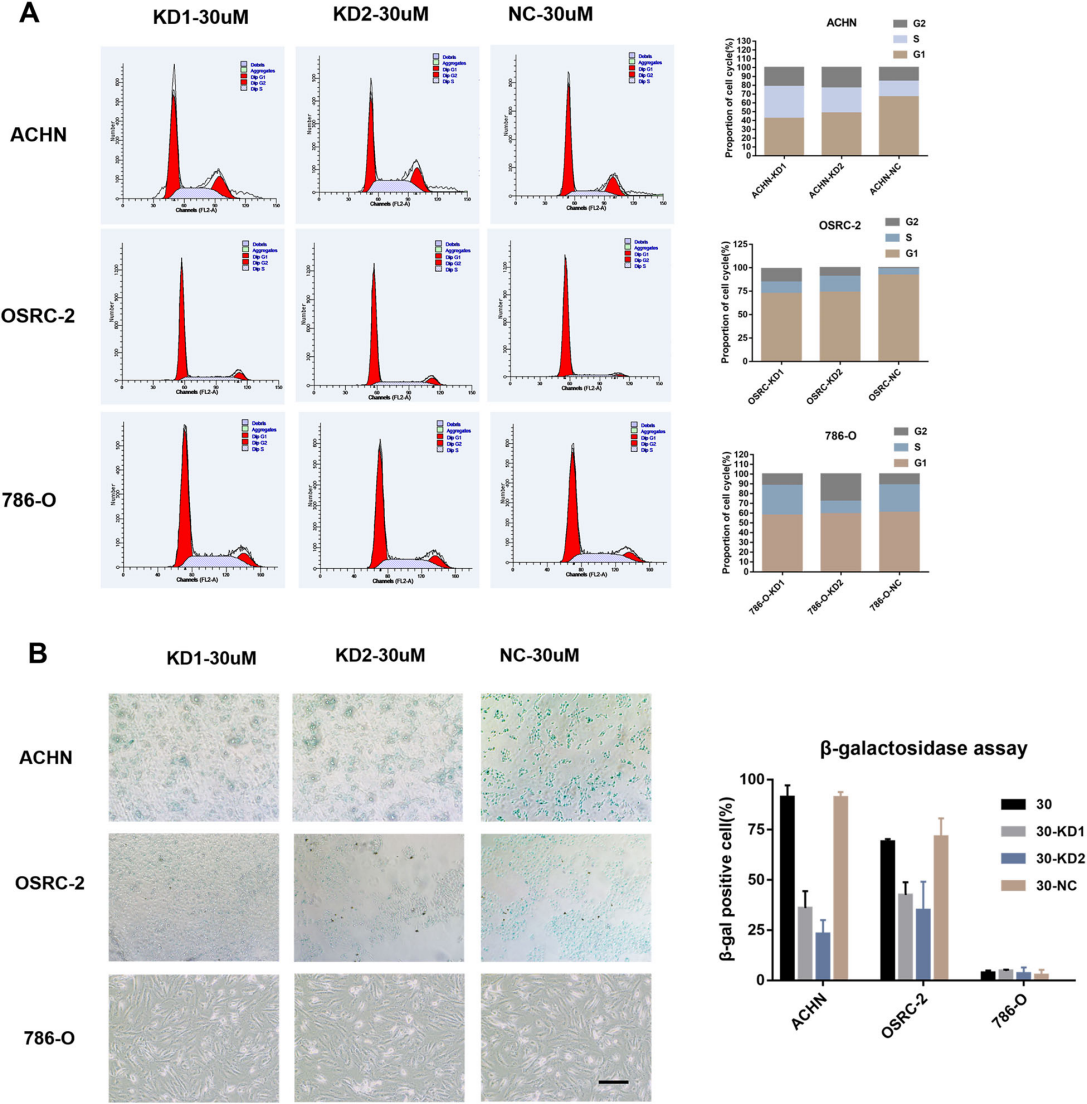
P53BER2 could mediate cell-cycle arrest and senescence in TP53-WT renal cancer cell lines. **A** The proportion of cell cycle of different RCC cell lines infected p53BER2-KD virus and vectors after nutlin-3 treatment. **B** The rate of senescence cell in different RCC cell lines infected p53BER2-KD virus and vectors after nutlin-3 treatment, scale bar, 100 μm. Results are shown as mean ± SD. ***P < 0.001 compared with control. **P < 0.01 compared with control. *P < 0.05 compared with control.

Cell-cycle arrest is one of the most noticeable biological outcomes of p53 activation in cell biology^32,33^. Therefore, we further analyzed whether p53BER2 affects cell phenotype by mediating changes in the cell cycle. Flow cytometry revealed that nutlin3 could cause the TP53-WT renal cancer cell line G1-arrest, but didn’t affect p53-MUT type renal cell carcinoma (Supplementary Fig. 3), which was consistent with previous research results. Interestingly, knockdown of p53BER2 reversed the nutlin3-mediated cell-cycle arrest TP53-WT renal cancer cell line (Fig. 4A). To confirm this finding, we first evaluated cellular entry into mitosis using phospho-H3 (ser10) staining. Cells stable expression amicroRNA targeting p53BER2 showed a significant decrease of phospho-H3 (ser10) compared with cells stable expressing NC vector after nutlin-3 treatment (Supplementary Fig. 4).

It is well known that p53 can also mediate cell senescence, so we further analyzed the effect of p53BER2 on cell senescence. We analyzed the senescence response of kidney cancer cell lines induced by nutlin3 by β-galactosidase assay. According to previous study^14^, we designed a test for the relationship between nutlin-3 concentration (24 h) and senescence. The results showed that nutlin3 could effectively induce a senescence reaction in TP53-WT renal cancer cells (Supplementary Fig. 5). At the same time, as we expected, knocking down p53BER2 effectively reversed the number of β-galactosidase-positive cells mediated by nutlin3, compared to the normal control group (Fig. 4B). P21 is a critical mediator of p53-induced senescence^34^. To further test if p21 was upregulated after Nutlin-3 in the cell lines tested with the KD of p53BER2 after Nutlin-3 treatment, we used nutlin-3 to treat OSRC-2 P53BER2-KD and Control groups and found that p21 is upregulated both in control and KD groups (Supplementary Fig. 1F). Even p21 is essential for senescence, from Fig. 4B, we could see downregulation of p53BER2 can partially rescue the senescence ratio of cancer cells, which suggested p53BER2 mediate senescence in a p21-independent way.

In conclusion, p53 can mediate the cell cycle and senescence response of the TP53-WT renal cancer cell line through p53BER2, thus affecting the development of renal cancer cell lines.

#### P53BER2 positively regulates cell cycle and DNA repair by regulating BRCA2

**Fig. 5:**
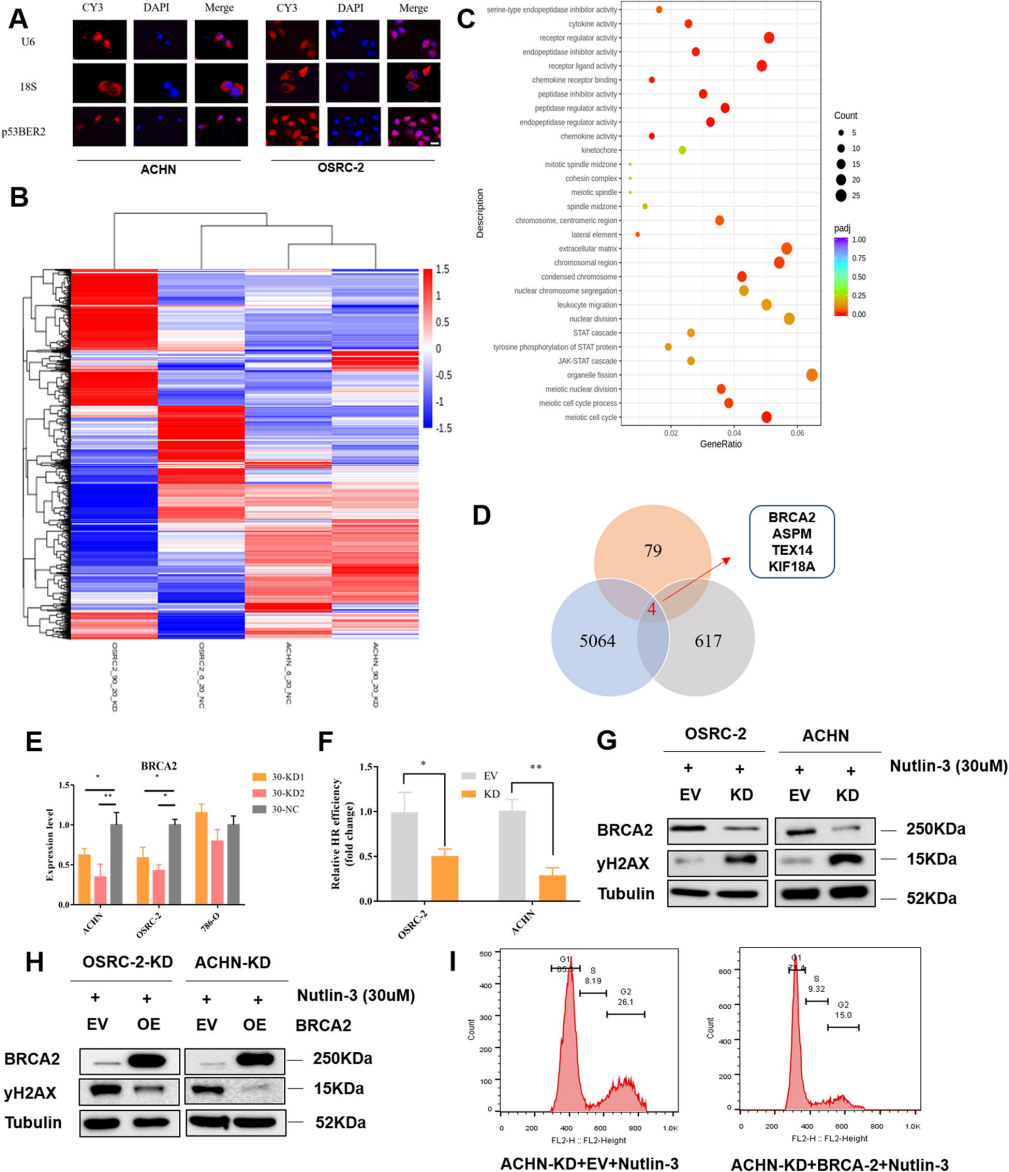
P53BER2 positively regulates cell cycle and DNA repair by mediating BRCA2. **A** The location of p53BER2 in RCC cell lines with confocal image, scale bar, 20 μm. **B** Heatmap representation of differentially expressed genes identified by RNA-Seq between P53BER-KD group and control group. **C** The Go terms enriched analysis of different biological processes in p53-KD and NC RCC line by RNA-sequencing. **D** Overlap of the differential genes of the two cell lines and the differential genes related to the cell-cycle pathway. **E** The BRCA2 expression level of different RCC cell lines infected p53BER2-KD virus and vectors after nutlin-3 treatment(30uM). **F** HR efficiency of ACHN and OSRC-2 after p53BER2 knockdown. **G** Western blot of yH2AX in p53BER2-KD and control cells after nutlin-3 treatments (30 μM). **H** Western blot of OSRC-2 and ACHN cells after transfected with BRCA2. **I** The proportion of cell cycle of ACHN-KD transfected BRCA2 after nutlin-3 treatment (30 μM). EV empty vector, KD p53BER2 knockdown, OE overexpression. Results are shown as mean ± SD. ***P < 0.001 compared with control. **P < 0.01 compared with control. *P < 0.05 compared with control.

Since the subcellular localization of lncRNA is associated closely with its biological function. We performed an RNA-fish experiment to find the location of P53ber2 in renal cancer. As expected, the results showed most p53BER2 was localized in the nucleus (Fig. 5A). Then, we performed RNA-sequencing by using OSRC-2-KD, ACHN-KD, and corresponding control cells. We found a lot of differently expressed genes between KD, and control groups, and these differently expressed genes could separate p53BER2-KD cells from control cells (Fig. 5B). Furthermore, we found that many differential genes were enriched in mitosis and cell cycle-related pathways (Fig. 5C). Most importantly, we found that most of the genes enriched in cell cycle-related pathways showed a downregulated trend (Supplementary Fig. 6). Therefore, we hypothesized that p53BER2 affects the cell cycle by affecting cell cycle-related genes. We further overlapped the differential genes of the two cell lines (OSRC-2:5064 and ACHN:617) and the differential genes related to the cell-cycle pathway (79). This resulted in a list of 4 potential downstream genes (Fig. 5D). We verified the mRNA levels of several genes in cell lines and found that downregulating p53BER2 caused BRCA2 downregulation (Fig. 5E), while others do not (Supplementary Fig. 7). According to previous studies, BRCA2 affects the cell-cycle process from G1 to S/G2 by affecting DNA repair. Furthermore, BRCA2 was known as a tumor suppressor in cancer by mediating DNA repair^35^. Therefore, we hypothesized that p53BER2 could influence cell cycle and DNA repair through BRCA2. Here, by using a PCR-based HR assay kit, we found HR efficiency in p53BER2-KD cells decreased significantly compared to the control group (Fig. 5F). Furthermore, we detected the expression of yH2AX (Phospho-Histone H2A.X-ser139), which is indicative of DNA damage and repair^36^, in OSRC-2/ACHN-KD and control after nutlin-3 treatment and found that yH2AX is upregulated in the p53BER2-KD group, which means p53BER2 could mediate BRCA2 to influence HR efficacy (Fig. 5G). Then, we overexpressed BRCA2 in P53BER2-KD OSRC-2 cell (Fig. 5H) lines and treat it with nutlin-3; the results showed BRCA2 could rescue the inhibition of the G1 cell-cycle arrest and HR efficiency caused by knocking down P53BER2 (Fig. 5I and Supplementary Fig. 7B, C). Furthermore, we chose 20 RNA samples from the previous cohort of patients to measure the expression of BRCA2, and the results showed the expression of BRCA2 was correlated significantly with the expression of p53BER2 (*P* < 0.01, Supplementary Table 2 and Table 3). The above evidence indicated that p53BER2 could mediate G1 cell-cycle arrest and increased HR efficacy by interacting BRCA2.

#### Downregulation of p53BER2 show resistance to nutlin-3 treatment in cell-derived xenografts (CDX)

**Fig. 6:**
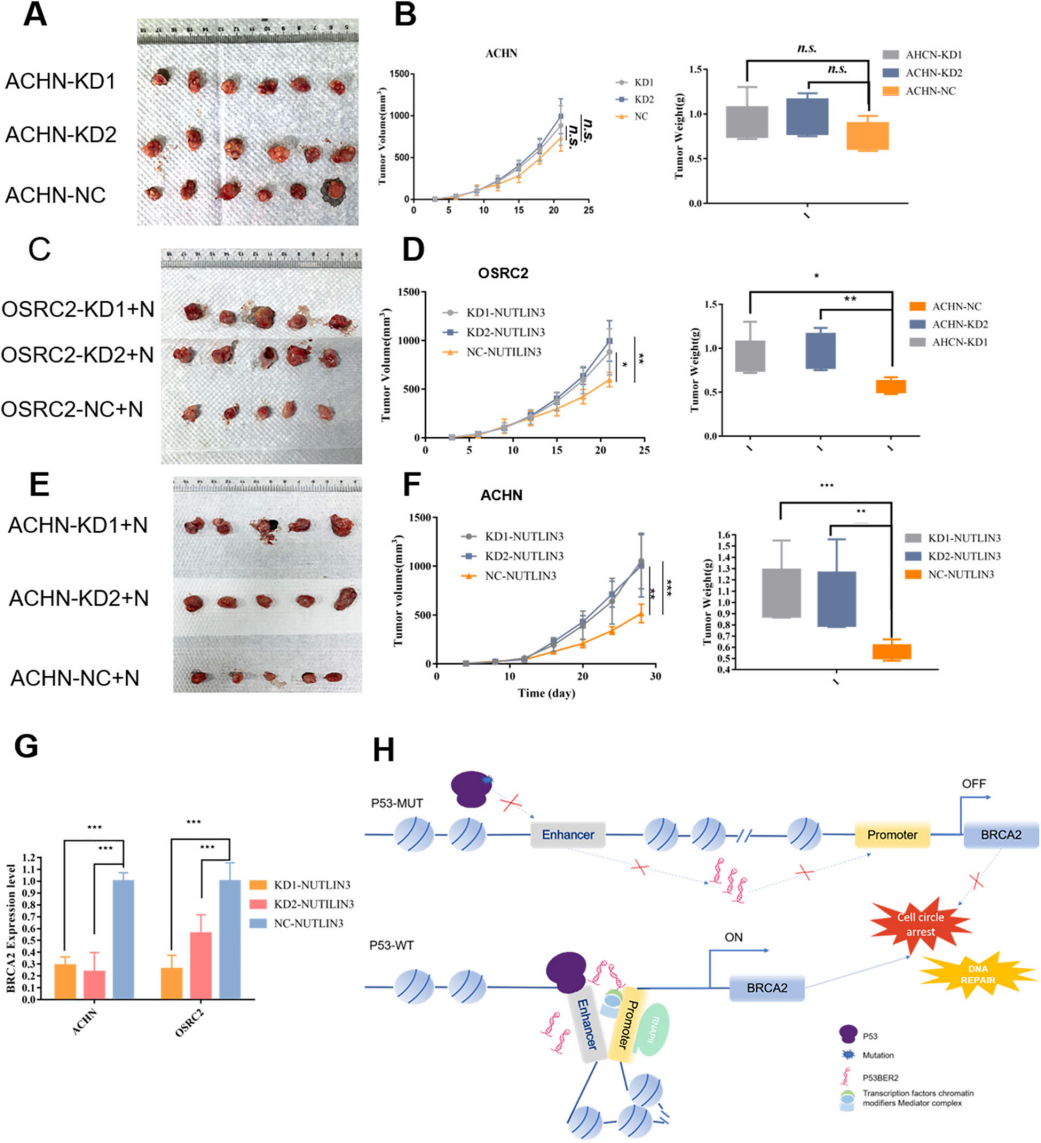
Downregulation of P53BER2 shows resistance to nutlin-3 treatment in cell-derived xenografts (CDXs). **A** Image of 18 xenografts formed by ACHN-KD1, KD2, and Control. **B** Growth curves of mice subcutaneous xenografts derived from ACHN-KD1, KD2, and Control cells (left panel). Tumor weights were statistically analyzed (right panel). **C** Image of 15 xenografts formed by OSRC-2-KD1, KD2, and Control with nutlin-3 treatment. **D** Growth curves of mice subcutaneous xenografts derived from OSRC-2-KD1, KD2, and Control cells with nutlin-3 treatment (left panel). Tumor weights were statistically analyzed (right panel). **E** Image of 15 xenografts formed by ACHN-KD1, KD2, and Control with nutlin-3 treatment. **F** Growth curves of mice subcutaneous xenografts derived from ACHN-KD1, KD2, and Control cells with nutlin-3 treatment (left panel). Tumor weights were statistical analyzed (right panel). **G** BRCA2 expression in mice subcutaneous xenograft. Results are shown as mean ± SD. ***P < 0.001 compared with control. **P < 0.01 compared with control. *P < 0.05 compared with control. **H** Schematic model of P53BER2-dependent regulation of P53 activity in p53-MUT or TP53-WT condition.

To understand the function of p53BER2 in vivo, we established the CDX model to compare the tumorigenic ability of the p53BER2 knockdown cell line and the control knockdown cell line. As shown in Fig. 6A, we can find that OSRC-2, which stably knocks down p53BER2, has stronger tumorigenic ability than OSRC-2 of the control group. The P53BER2 knockdown group had a faster tumorigenic capacity relative to the control group (Fig. 6B). At the same time, the tumor weight of the p53BER2 knockdown group was slightly higher than that of the control group (*P* < 0.05) (Fig. 6C). Further, we injected intraperitoneal injection of the p53BER2-KD group and NC group with nutlin-3, and the results showed that the p53BER2 knockdown group was small. Xenograft formed by RCC infected with p53BER2-KD were significantly resistant to nutlin-3 mediated anti-tumor effect compared to control mice (Fig. 6D), with faster growth rates (Fig. 6E) and larger tumors (Fig. 6F). Moreover, BRCA2 expression in mice subcutaneous xenograft showed consistency with the expression with p53BER2 (Fig. 6G), which suggested BRCA2 could also be regulated by p53BER2 in vivo.

## Discussion

The function of p53 in RCC remains unclear. The traditional function of p53 seems restricted in kidney cancer. Our study aimed to find the new potential mechanism about the p53 pathway in RCC and tried to reactivate the function of p53 in kidney cancer. From the first identification, we found that p53BER2 expression was downregulated in renal cancer tissues and cell lines and could specifically express in TP53-WT renal cancer cell lines. Then we found downregulation of p53BER2 could reverse the nutlin-3-induced cytotoxic effect in TP53-WT cell lines. Further, to explore the relationship between p53BER2 and p53-related function, we found the downregulation of p53BER2 could reverse nutlin-3-induced G1-arrest and senescence in TP53-WT cell lines. What is more, the downregulation of p53BER2 showed resistance to nutlin-3 treatment for tumor treatment in the CDX model. Additionally, we found the p53BER2 could regulate BRCA2 in vitro and vivo; further experiment showed p53BER2 could induce cell-cycle arrest and DNA repair by mediating BRCA2. Therefore, our study revealed that enhancer RNA-p53BER2 could mediate p53 function in RCC, which provided a novel perspective for p53 research in RCC.

P53 research has caught much attention in oncology, including kidney cancer. Although VHL is the most critical mutation in kidney cancer, p53 still has a vital role in the tumorigenesis of kidney cancer^37^. Some studies indicate that p53 is highly expressed in kidney cancer tissues, indicating that p53 may have a cancer-promoting effect on kidney cancer^11,38^. However, through the MDM2 inhibitor method, studies have found that p53 activation can effectively inhibit the proliferation of renal cancer cell lines^14,39^, which suggests that p53 could also has an essential role in kidney cancer, and new related signaling pathways may need to explore. Recently, studies have found that as a transcription factor, p53 can activate the transcription of coding genes, and the transcription of non-coding genes^22,40^. Among them, the most interesting is the application of eRNAs in p53 pathway. P53 could also target some enhancer sequences and induce the corresponding eRNAs expression, further activating adjacent genes^22^. More importantly, these eRNAs were proven to be functional in cell physiology. Therefore, we wanted to figure out whether p53-related eRNAs also have a role in kidney cancer. Our results indicate that p53BER2 expression is significantly downregulated in renal cancer clinical specimens. Further in vivo and in vitro experiments found that p53BER2 can effectively mediate p53’s functions in the cell cycle and senescence. The above results show that p53-related RNA can have a vital role in renal cancer occurrence and development.

At present, literature is showing that p53-related enhancer RNA can effectively regulate the expression of downstream genes^22,41^. Besides being regulated by a chromosomal loop, p53-associated enhancer RNA can also be regulated by long non-coding RNA^41^. In our research, by RNA-sequencing, we found that knockdown of p53BER2 can cause downregulation of cell cycle-related downstream genes. Among them, through experimental verification, we found that knocking down p53BER2 can repress BRCA2 expression levels. Furthermore, this result was verified in nude mouse experiments. BRCA2 is a tumor suppressor, which has an integral part in cell physiology by promoting DNA replication and DNA double-strand breaks (DSBs)^42^. Studies show that the loss of BRCA2 can trigger a significant reduction in the replication fork process and affect DNA replication^43,44^. At this time, in tumor cells, replication stress and DNA damage tolerance will allow the cells to survive the loss of BRCA2 and further stimulate the cell’s tumorigenesis potential^45,46^. Therefore, patients who carry BRCA2 germline mutations are much susceptible to breast cancer and ovarian cancers^35,47,48^. More importantly, a mouse model has shown that disruption of the p53 pathway is crucial for BRCA2-related breast cancer^49^. Furthermore, p53 can affect the expression of BRCA2 by interacting with the BRCA2 promoter in response to DNA damage^50^. Consistent with these studies, we found a positive correlation between P53BER2 and BRCA2, suggesting that p53BER2 may mediate cell cycle-related phenotypic effects through BRCA2 (Fig. 6H).

Our work’s highlight was the p53BER2 could function in the RCC, which is the first time to discuss eRNAs in RCC. Compared with traditional coding-gene related analysis, we paid attention to p53 relative eRNAs and tried to figure out its function in RCC, which partly explained the function of p53 in RCC. Additionally, MDM2 inhibitor-nutlin3 can effectively repress RCC’S proliferation in vitro and vivo, which might be able to serve as an anti-tumor reagent potentially in TP53-WT RCC patient. Furthermore, we found a new target of p53BER2-BRCA2 and reveal a new function of p53BER2 in cell cycle and DNA repair by mediating BRCA2. What is more, the p53BER2 expression level is deficient in most RCC samples, which means this pathway is inhibited in kidney cancer; if we could reactivate this pathway in RCC cell lines, it might be possible to treat the RCC patients by activating the p53-p53BER2 pathway. However, there are a few limitations that need to address. Firstly, the complete sequence of p53BER2 still cannot be captured, which might need some advanced technique to make it come true, such as global nuclear run-on sequencing (GRO-seq). Then, the incidence of TP53 mutation in RCC is less than the other tumors, but p53BER2 still maintains a low expression level, which might be caused by some “wrong” p53 or detect in the p53 pathway in RCC. The relationship between p53BER2 and “wrong” p53 would be excited and worth digging out. Even so, we still believe our study provided a novel approach in RCC and p53-related study.

Under some conditions, enhancers could transcript some specific RNA, which can interact with other genes and regulate it. In our study, we showed that enhancer RNA-p53BER2 could partly mediate the function of p53 in TP53-WT RCC, in vitro and vivo. Moreover, we found tumor suppressor-BRCA2 had a positive correlation with p53BER2; further experiments showed the p53BER2 could induce cell-cycle arrest and DNA repair by mediating BRCA2. In conclusion, our study found an interesting association in RCC and provided a novel way to study RCC and p53 research.

## Supplementary information

Supplementary tables and figure legends

Supplementary figure 1

Supplementary figure 2

Supplementary figure 3

Supplementary figure 4

Supplementary figure 5

Supplementary figure 6

Supplementary figure 7

## Data Availability

All data generated or analyzed during this study are included in this article.
