# Supplementary material for: Cell-cycle arrest and senescence in TP53-wild type renal carcinoma by enhancer RNA-P53-bound enhancer regions 2 (p53BER2) in a p53-dependent pathway
Source: Cell Death Dis. 2021 Jan 5;12(1):1. doi: 10.1038/s41419-020-03229-8 (PMC7791070; doi:10.1038/s41419-020-03229-8)
Supplement: Supplementary file 1 — Supplementary tables and figure legends [file 41419_2020_3229_MOESM1_ESM.docx]

**Supplementary table 1** The main features of the renal carcinoma for included patients.

| Pt No. | Gender | T stage | Grade | -ΔΔCT | Pt No. | Gender | T stage | Grade | -ΔΔCT |
| --- | --- | --- | --- | --- | --- | --- | --- | --- | --- |
| 1 | Male | PT1a | G2 | -13.15 | 43 | Male | pT1a | G1 | -3.312183 |
| 2 | Male | pT1A | G2 | -10.9234 | 44 | Male | PT1b | G1 | -3.23 |
| 3 | Female | pT1A | G2 | -10.73311 | 45 | Male | pT1B | G2 | -3.127722 |
| 4 | Male | PT1a | G2 | -10.39 | 46 | Male | PT1b | G1 | -2.99 |
| 5 | Male | PT1a | G2 | -10.01 | 47 | Male | pT3A | G2 | -2.772602 |
| 6 | Female | pT1b | G2 | -9.322899 | 48 | Male | pT3A | G2 | -2.640511 |
| 7 | Male | PT1a | G1 | -9.18 | 49 | Male | pT1A | G2 | -2.552362 |
| 8 | Male | PT1a | G1 | -8.620087 | 50 | Female | PT1b | G2 | -2.49 |
| 9 | Female | PT3a | G3 | -8.4 | 51 | Male | NA | NA | -2.410276 |
| 10 | Male | pT1b | G2 | -8.107092 | 52 | Male | pT3B | G4 | -2.379208 |
| 11 | Female | pT1B | G3 | -8.102309 | 53 | Male | pT1a | G1 | -2.318651 |
| 12 | Male | pT1a | G1 | -7.989935 | 54 | Female | pT1A | G2 | -2.312155 |
| 13 | Female | PT1b | G1 | -7.962685 | 55 | Male | PT1a | G2 | -2.31 |
| 14 | Female | pT3A | G2 | -7.55793 | 56 | Male | pT1b | G1 | -2.002304 |
| 15 | Male | PT1a | G2 | -7.43 | 57 | Male | pT1a | G1 | -1.420927 |
| 16 | Male | pT1a | G2 | -7.391418 | 58 | Male | pT1A | G2 | -1.356632 |
| 17 | Male | PT1a | G2 | -7.29 | 59 | Female | PT2 | G1 | -1.29 |
| 18 | Male | PT1a | G1 | -6.8 | 60 | Male | pT3a | G2 | -1.264034 |
| 19 | Female | pT1B | G2 | -6.572317 | 61 | Female | pT1b | G2 | -1.136057 |
| 20 | Male | pT1a | G2 | -6.329939 | 62 | Male | PT1b | G1 | -1.11 |
| 21 | Female | pT1B | G2 | -6.219675 | 63 | Female | pT1A | G2 | -1.022832 |
| 22 | Male | pT1B | G1 | -6.19392 | 64 | Male | PT1a | G1 | -0.94 |
| 23 | Female | Pt2b | G2 | -6.12 | 65 | Male | PT1b | G1 | -0.74 |
| 24 | Male | pT1a | G1 | -5.963026 | 66 | Female | pT1a | G1 | -0.721066 |
| 25 | Male | pT1B | G2 | -5.664587 | 67 | Male | pT1a | G3 | -0.410864 |
| 26 | Female | PT1a | G1 | -5.604706 | 68 | Male | pT1A | G1 | 0.027439 |
| 27 | Male | PT1b | G2 | -5.507671 | 69 | Male | PT1a | G1 | 0.09 |
| 28 | Male | pT1B | G2 | -5.459263 | 70 | Female | PT1b | G2 | 0.297503 |
| 29 | Male | pT1A | G2 | -5.416159 | 71 | Male | PT1b | G3 | 0.99 |
| 30 | Female | PT1a | G1 | -5.28 | 72 | Male | pT1B | G2 | 1.042088 |
| 31 | Male | pT3B | G3 | -5.197598 | 73 | Female | PT1b | G2 | 1.48 |
| 32 | Male | pT3a | G2 | -5.190809 | 74 | Female | pT3B | G2 | 1.497572 |
| 33 | Female | pT1A | G1 | -4.893976 | 75 | Male | PT1a | G1 | 1.504543 |
| 34 | Female | pT1B | G2 | -4.833538 | 76 | Male | pT1B | G2 | 1.84606 |
| 35 | Male | pT1A | G3 | -4.703754 | 77 | Male | pT3B | G3 | 1.888748 |
| 36 | Male | pT1A | G1 | -4.689569 | 78 | Male | pT1A | G2 | 1.948803 |
| 37 | Male | pT1A | G2 | -4.271528 | 79 | Female | PT3b | G2 | 2.47 |
| 38 | Male | pT3A | G2 | -4.208868 | 80 | Male | PT1b | G2 | 2.67 |
| 39 | Female | pT1B | G1 | -4.148299 | 81 | Male | PT1a | G2 | 3.09 |
| 40 | Male | pT3a | G2 | -3.976625 | 82 | Male | PT1a | G2 | 3.13 |
| 41 | Male | PT1a | G3 | -3.802607 | 83 | Male | PT1b | G1 | 4.67 |
| 42 | Male | pT1b | G1 | -3.65 | 84 | Female | PT1b | G2 | 5.62 |

Pt No. patient number; M male; F female; Grade: Fuhrman Grade. CT: cycle threshold. NA: not available.

**Supplementary table 2 The expression of BRCA2 and p53BER2 in 20 RCC samples.**

| Pt No. | Gender | T stage | Grade | -ΔΔCT/P53BER2 | -ΔΔCT/BRCA2 |
| --- | --- | --- | --- | --- | --- |
| 1 | Male | PT1a | G2 | -13.15 | -7.323453 |
| 2 | Male | pT1A | G2 | -10.9234 | -9.32 |
| 3 | Female | pT1A | G2 | -10.73311 | -6.570138 |
| 4 | Male | PT1a | G2 | -10.39 | -10.598274 |
| 5 | Male | PT1a | G2 | -10.01 | -5.023 |
| 20 | Male | pT1a | G2 | -6.329939 | -2.324542 |
| 21 | Female | pT1B | G2 | -6.219675 | -4.512345 |
| 22 | Male | pT1B | G1 | -6.19392 | -2.172112 |
| 23 | Female | Pt2b | G2 | -6.12 | -3.581793 |
| 24 | Male | pT1a | G1 | -5.963026 | -3.404203 |
| 50 | Female | PT1b | G2 | -2.49 | 0.892418 |
| 51 | Male | NA | NA | -2.410276 | -0.569156 |
| 52 | Male | pT3B | G4 | -2.379208 | -0.355984 |
| 53 | Male | pT1a | G1 | -2.318651 | 2.3 |
| 54 | Female | pT1A | G2 | -2.312155 | -0.120473 |
| 80 | Male | PT1b | G2 | 2.67 | -1.459012 |
| 81 | Male | PT1a | G2 | 3.09 | 3.84 |
| 82 | Male | PT1a | G2 | 3.13 | 2.19 |
| 83 | Male | PT1b | G1 | 4.67 | 3.56 |
| 84 | Female | PT1b | G2 | 5.62 | 6.89 |

Pt No. patient number; M male; F female; Grade: Fuhrman Grade. CT: cycle threshold. NA: not available.

**Supplementary table 3 Correlation of BRCA2 and P53BER2 in 20 RCC samples.**

|  | BRCA2 low | BRCA2 high | Total | χ^2^ | P-value |
| --- | --- | --- | --- | --- | --- |
| P53BER2 low | 13 | 2 | 15 |  |  |
| P53BER2 high | 1 | 4 | 5 |  |  |
| Total | 14 | 6 | 20 | 6.508 | 0.007 |

**Supplementary table 4** cDNA sequences of vectors and relative primers used in this research.

| Vector Names | Synthetic Sequences |
| --- | --- |
| Artificial micro-RNA 1 | CTGGAGGCTTGCTGAAGGCTGTATGCTGGCTGGACACTGGGTAAATCAGAGTTTTGGCCACTGACTGACTCTGATTCCAGTGTCCAGCCAGGACACAAGGCCTGTTACTAGCACTCACATGGAACAAATGGC |
| Artificial micro-RNA 2 | CTGGAGGCTTGCTGAAGGCTGTATGCTGGCACAGATTCCGTGTAAATACTGTTTTGGCCACTGACTGACAGTATTTCGGAATCTGTGCCAGGACACAAGGCCTGTTACTAGCACTCACATGGAACAAATGGC |
| NC-pZDonor-hef1a-Puro-control miRNA (FF5) | CTGGAGGCTTGCTGAAGGCTGTATGCTGTAATTGTCAAATCAGAGTGCTTGTTTTGGCCACTGACTGACAAGCACTATTTGACAATTACAGGACACAAGGCCTGTTACTAGCACTCACATGGAACAAATGGC |
| P53BER2-site | ATGGTCATTCACCATGCAACCTCTGCTGTTAACAGATTTCAGACTGTAATCTTTTGTTTCTTCTTTCTAATCCTGCACTTCCTCTGACTGTTCCATTTTGGTTCCGAGCAAGCAGGCCGCTTGTGTCTGAATCTGGCCAATCCTGAAATCTCTGCTTGGCTTTGTCAGACAAGTTCAGGCATGCACAGACATGTCCATGAGTTTCTCAGAAGGTTGACTCTGCCAAAGCCAGAACACCACTCCCCCACTTTCCACTGGGTCCTGTGCTCCAAGCTATTGGACATAAGTTTGTGCTTTCGAAACTTCAGTATTTACACGGAATCTGTG |
| pLX313-TP53-WT | Addgene, 118014 |
| pLX313-TP53-P278A | Addgene, 118015 |
| SI-TP53-1 | GUGCAGCUGUGGGUUGAUU (J-003329-15, DHARMACON) |
| SI-TP53-2 | GCAGUCAGAUCCUAGCGUC (J-003329-16, DHARMACON) |
| Primer name | Sequences |
| p53BER2-F | CCAGTGGAAAGTGGGGGAGT |
| P53BER2-R | CCTGAAATCTCTGCTTGGCTTTG |
| p53BER4-F | TGGCACTGGGCTTAGGTCTTTT |
| P53BER4-R | CCCACAAGGGCTCTCAAGTTC |
| GAPDH-F | CGCTCTCTGCTCCTCCTGTTC |
| GAPDH-R | ATCCGTTGACTCCGACCTTCAC |
| BRCA2-F | CACCCACCCTTAGTTCTACTGT |
| BRCA2-R | CCAATGTGGTCTTTGCAGCTAT |
| PUMA-F | GACCTCAACGCACAGTACGAG |
| PUMA-R | AGGAGTCCCATGATGAGATTGT |
| P21-F | TGTCCGTCAGAACCCATGC |
| P21-R | AAAGTCGAAGTTCCATCGCTC |
| PAPPA-F | TGCCGAGAGAATAAGCACAAGG |
| PAPPA-R | GGTGGAGGTGGGTCACAGG |
| Antibody name | Brand |
| α-Tubulin (DM1A) Mouse mAb | α-Tubulin (DM1A) Mouse mAb #3873 |
| Monoclonal Anti-Vinculin antibody | Sigma-Aldrich, V9131 |
| Anti-P53-MOUSE | Abcam-ab26 p53 monoclonal antibody |
| Secondary antibody | ProteinFind Goat Anti-Rabbit/Mouse IgG(H+L), HRP conjugate, 1:1000  dilution, #HS201, TransGen, Beijing, China |
| Anti-Histone H3(phospho S10)-rabbt | Abcam Chip Grade ab177218 |
| Anti-rabbit IgG | Goat Anti-rabbit IgG H&L (Alexa Fluor 488) ab150077 |
| P21 Waf1/Cip1 (12D1) Rabbit mAb | Cell signaling technolgoy #2947 |
| Anti- Brca2 | Bethyl Laboratories/ A300-005A-T |
| Phospho-Histone H2A.X (Ser139) (20E3) Rabbit mAb | Cell signaling technolgoy #9718 |

Note: F, forward primer; R, reverse primer.

**Supplementary figure legends**

Supplementary figure 1. Cell viability of RCC cell lines with nutlin3 treatment. A: The cell viability curves of RCC cell lines with nutlin3 treatment. B: IC50 value of different RCC cell lines infected p53BER2-KD virus and vectors after nutlin-3 treatment. C: The relative P21 expression level in RCC lines after nutlin-3 treatment. D: The relative PUMA expression level in RCC lines after nutlin-3 treatment. E: The relative PAPPA expression level in RCC lines after nutlin-3 treatment. F: P21 could be induced in OSRC-2 p53BER2 KD and control group after nutlin-3 treatments. ***P < 0.001 compared with control. ** P < 0.01 compared with control. * P < 0.05 compared with control. NS, not significantly.

Supplementary figure 2. A: Western blot of H12PP-WT-TP53 after transfected with si-TP53. B: Relative p53BER2 expression level of H12PP-WT-TP53 after transfected with si-TP53. C: The dual-luciferase activity of H1299/TP53-WT/ TP53-P278A after transfected with “p53BER2 reporter” and “NC reporter”. D：The dual-luciferase activity of H1299 TP53-P278A after PRIMA-1-met treatment. E: The apoptotic rate of different RCC cell lines or stable cell lines after nulin-3 treatment. EV: empty vector.NC: negative control. Results are shown as mean ± SD. ***P < 0.001 compared with control. ** P < 0.01 compared with control. * P < 0.05 compared with control. NS, not significantly.

Supplementary figure 3. The proportion of cell cycle of different RCC cell after nutlin-3 treatment.

Supplementary figure 4. H3ser10 staining with immunofluorescence showed the process of cell cycle in different RCC cell lines or stable cell lines after nulin-3 treatment. Scale bar, 20 μm.

Supplementary figure 5. The rate of senescence cell in different RCC cell lines after nutlin-3 treatment. Scale bar, 100 μm.

Supplementary figure 6. The overall expression level of several biological process related gene between p53-KD and NC RCC line from RNA-sequencing data.

Supplementary figure 7. A: Expression level of potential target genes in ACHN cell lines infected p53BER2-KD or vectors lentivirus. B: HR efficiency of ACHN and OSRC-2 transfected BRCA2 after nutlin-3 treatment (30uM). C: The proportion of cell cycle of OSRC-2 KD transfected BRCA2 after nutlin-3 treatment (30uM). EV: empty vector. Results are shown as mean ± SD. ***P < 0.001 compared with control. ** P < 0.01 compared with control. * P < 0.05 compared with control. NS, not significantly.

Supplementary table. The detailed information was shown in the file- “supplementary table”.
