# Supplementary figures and images for: Cell-cycle arrest and senescence in TP53-wild type renal carcinoma by enhancer RNA-P53-bound enhancer regions 2 (p53BER2) in a p53-dependent pathway
Source: Cell Death Dis. 2021 Jan 5;12(1):1. doi: 10.1038/s41419-020-03229-8 (PMC7791070; doi:10.1038/s41419-020-03229-8)

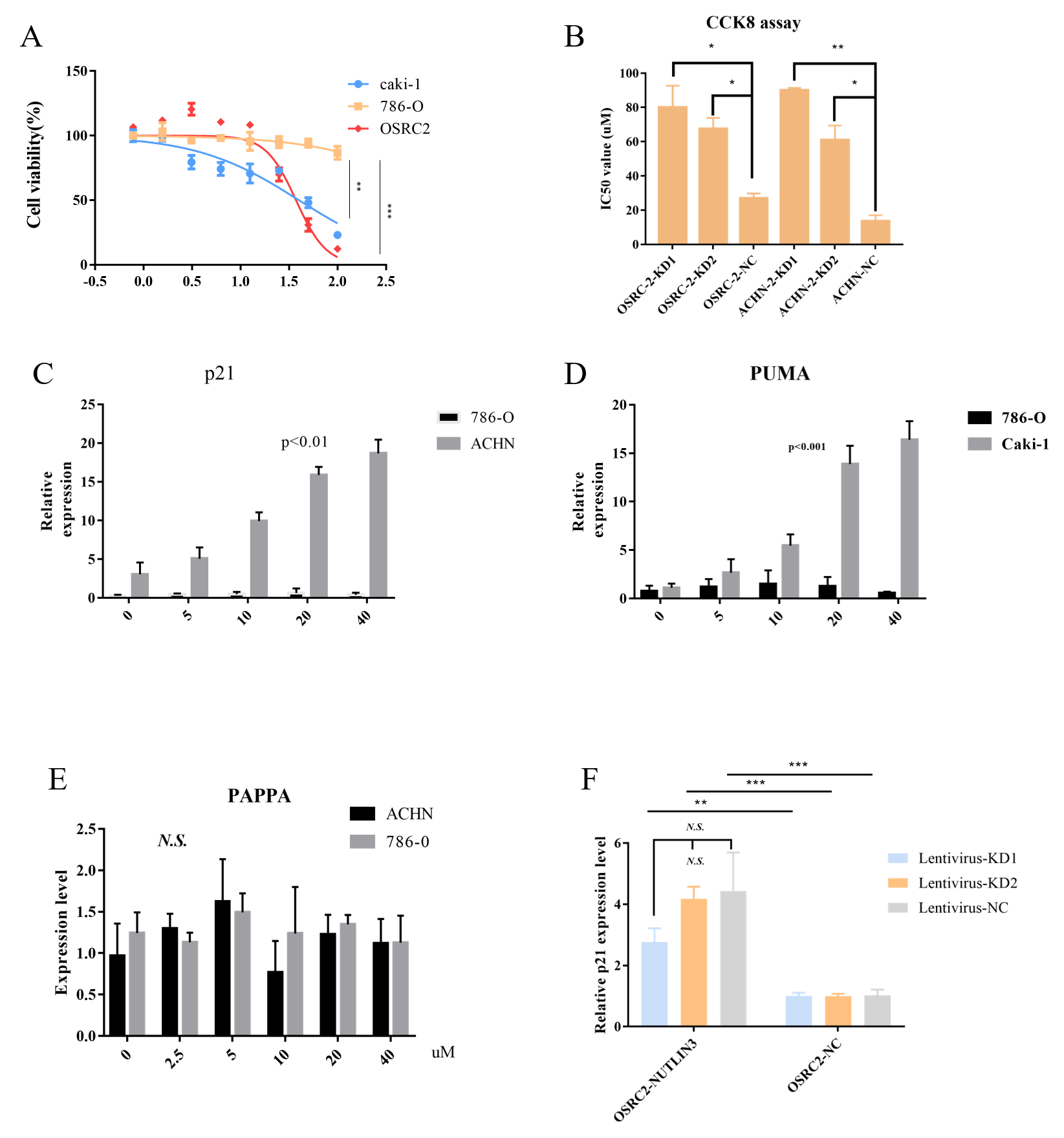

Supplement: Supplementary file 2 — Supplementary figure 1 [file 41419_2020_3229_MOESM2_ESM.tif]

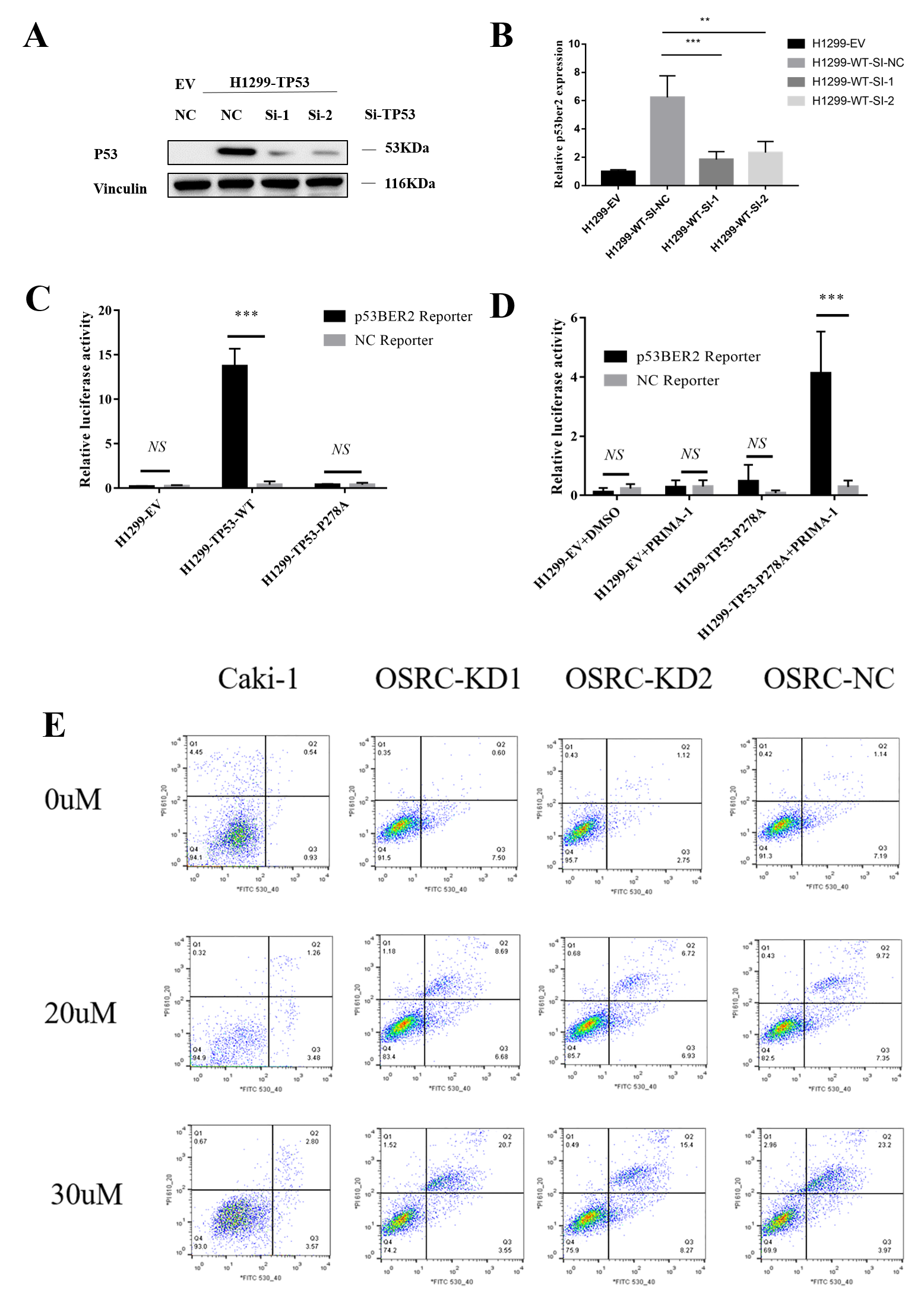

Supplement: Supplementary file 3 — Supplementary figure 2 [file 41419_2020_3229_MOESM3_ESM.tif]

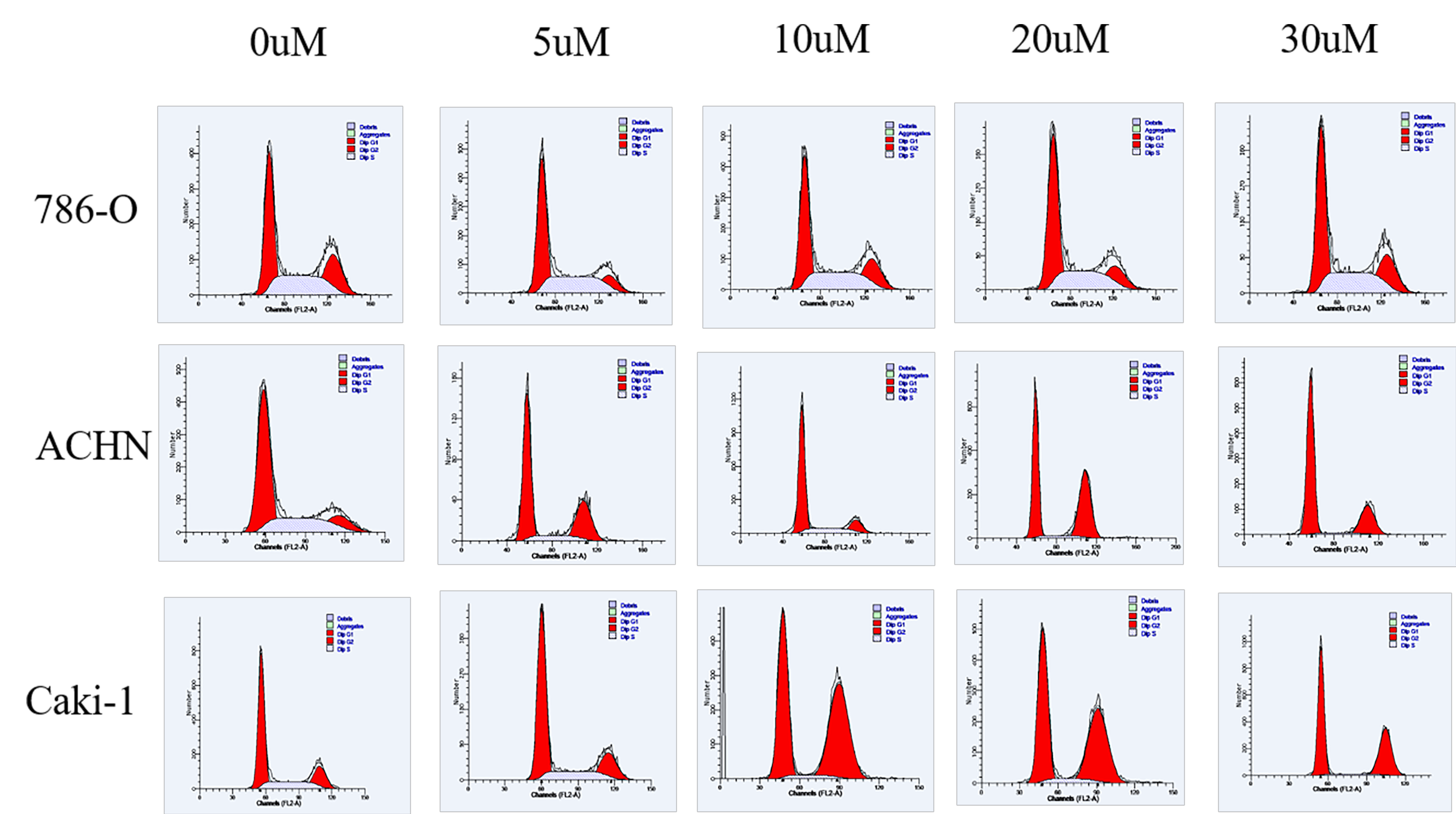

Supplement: Supplementary file 4 — Supplementary figure 3 [file 41419_2020_3229_MOESM4_ESM.tif]

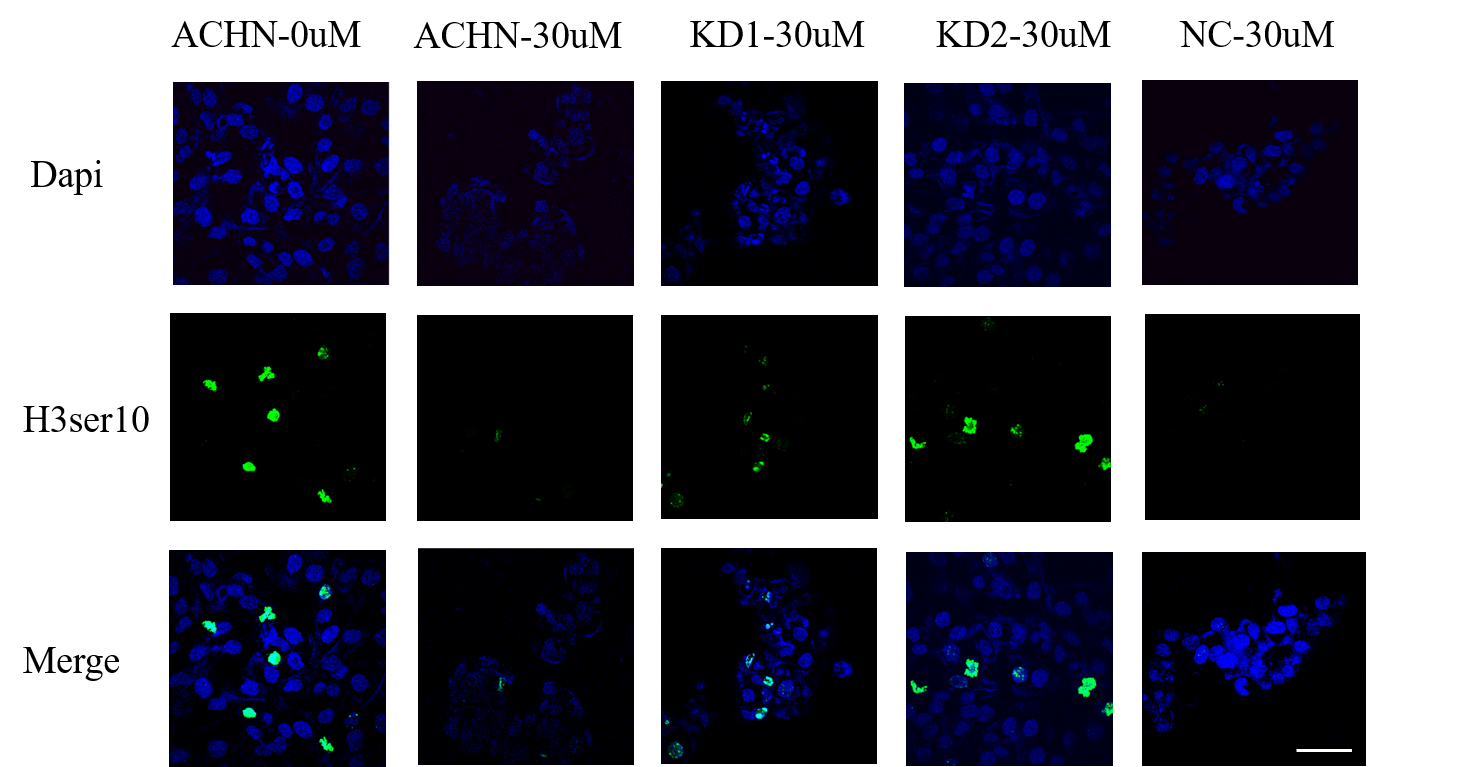

Supplement: Supplementary file 5 — Supplementary figure 4 [file 41419_2020_3229_MOESM5_ESM.tif]

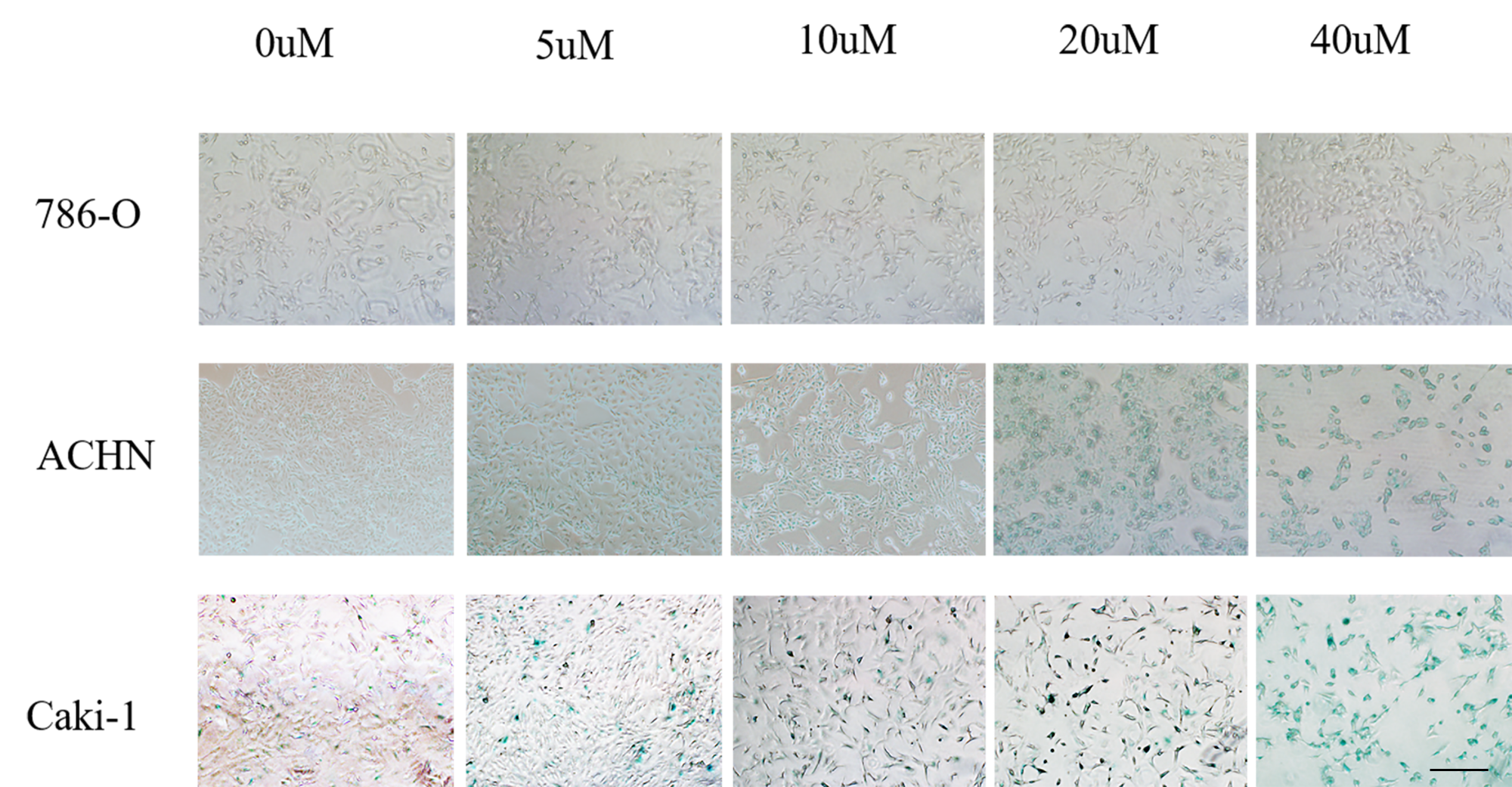

Supplement: Supplementary file 6 — Supplementary figure 5 [file 41419_2020_3229_MOESM6_ESM.tif]

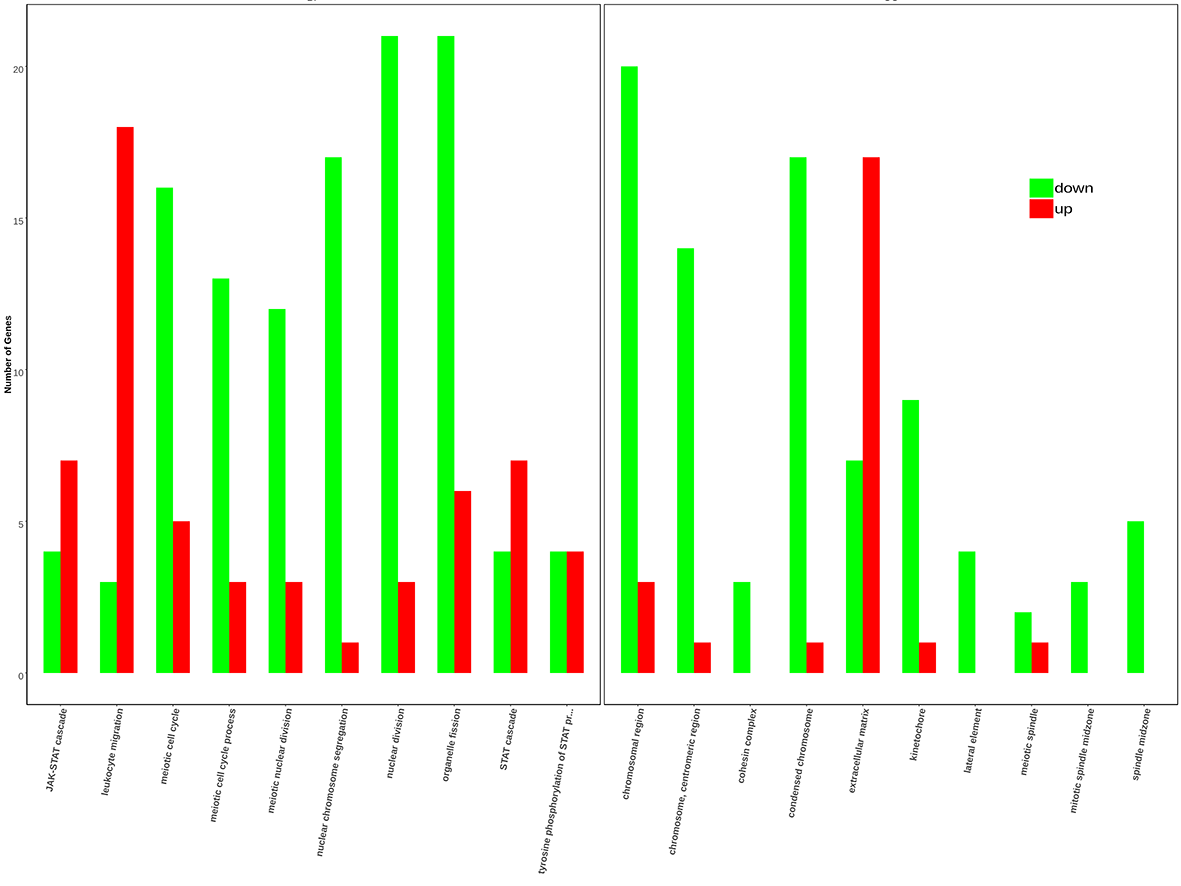

Supplement: Supplementary file 7 — Supplementary figure 6 [file 41419_2020_3229_MOESM7_ESM.tif]

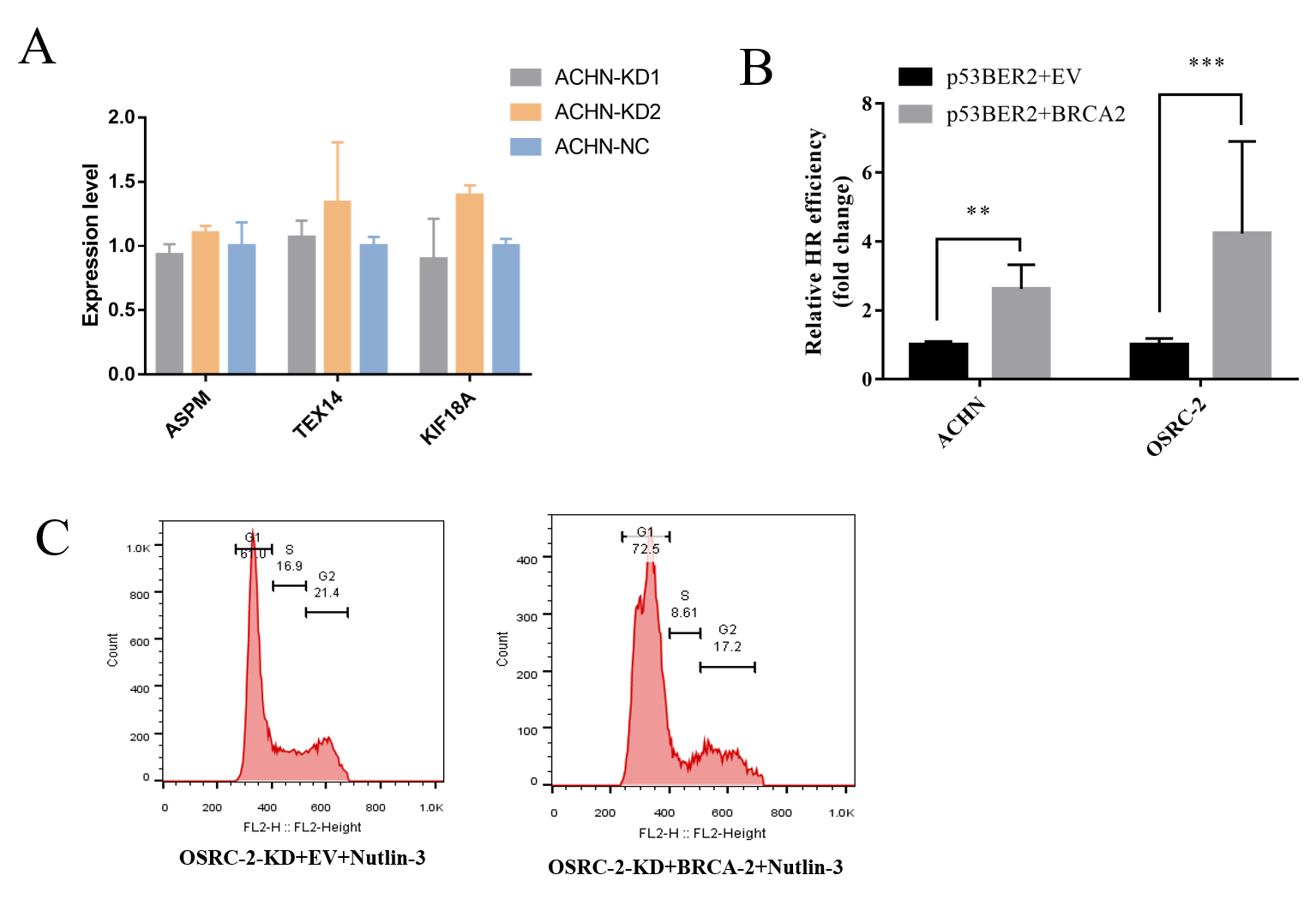

Supplement: Supplementary file 8 — Supplementary figure 7 [file 41419_2020_3229_MOESM8_ESM.tif]
